# Social insecurity in relation to orbitofrontal activity in patients with eating disorders: a near-infrared spectroscopy study

**DOI:** 10.1186/1471-244X-14-173

**Published:** 2014-06-12

**Authors:** Hiroto Katayama, Kunihiro Kohmura, Satoshi Tanaka, Miho Imaeda, Naoko Kawano, Yukihiro Noda, Kazuo Nishioka, Masahiko Ando, Branko Aleksic, Tetsuya Iidaka, Norio Ozaki

**Affiliations:** 1 Department of Psychiatry, Nagoya University Graduate School of Medicine, 65 Tsurumai-cho, Showa-ku, Nagoya, Aichi-ken 466-8550, Japan; 2 Department of Psychiatry, Nagoya University Hospital, 65 Tsurumai-cho, Showa-ku, Nagoya, Aichi-ken 466-8550, Japan; 3 Division of Clinical Science and Neuropsychopharmacology, Graduate School of Pharmacy, Meijo University, 150 Yagotoyama, Tenpaku-ku, Nagoya, Aichi-ken 468-8503, Japan; 4 The Academic Frontier Project for Private Universities, Comparative Cognitive Science Institutes, Meijo University, 1-501 Shiogamaguchi, Tenpaku-ku, Nagoya, Aichi-ken 468-8502, Japan; 5 Center for Advanced Medicine and Clinical Research, Nagoya University Hospital, Nagoya, Aichi-ken 466-8550, Japan

**Keywords:** Anorexia nervosa, Extremely low body weight, Near-infrared spectroscopy, Social isolation

## Abstract

**Background:**

Functional neuroimaging techniques are widely used to elucidate changes in brain activity, and various questionnaires are used to investigate psychopathological features in patients with eating disorders (ED). It is well known that social skills and interpersonal difficulties are strongly associated with the psychopathology of patients with ED. However, few studies have examined the association between brain activity and social relationships in patients with ED, particularly in patients with extremely low body weight.

**Methods:**

In this study, 22-channel near-infrared spectroscopy was used to quantify regional hemodynamic changes during a letter fluency task (LFT) in 20 female patients with ED with a mean body mass index of 14.0 kg/m^2^and 31 female controls (CTLs). Symptoms were assessed using the Eating Disorder Inventory-2 and Beck Depression Inventory. We hypothesized that frontal activity in patients with ED would be lower than in CTLs and would show different correlations with psychopathological features compared with CTLs.

**Results:**

The LFT performance and score on the social insecurity subscale of the Eating Disorder Inventory-2 were significantly higher in the ED group than in the CTL group. The mean change in oxygenated hemoglobin (oxy-Hb) in bilateral frontal regions during the LFT was significantly smaller in the ED group than in the CTL group. Social insecurity score was positively correlated with the concentration of oxy-Hb in the bilateral orbitofrontal cortex in the ED group but not in the CTL group.

**Conclusions:**

These results suggest that activity of the orbitofrontal cortex is associated with social insecurity and disturbed in patients with ED. Therefore, disturbed orbitofrontal cortex activity may underlie the lack of insight and social isolation that is characteristic of patients with ED.

## Background

Anorexia nervosa (AN) is an eating disorder (ED) characterized by food restriction, inappropriate eating habits, obsession with having a thin figure, an irrational fear of weight gain, and a distorted body self-perception [1]. AN is increasingly recognized as a serious disease that affects many young individuals. However, its etiology is complex and treatment effect is limited [1]. Problems with homeostasis, drive, and self-regulation are biological factors that are known to be associated with AN [1]. Although findings of magnetic resonance imaging studies of subjects with AN are inconsistent [2], other functional neuroimaging modalities, including single-photon emission tomography, positron emission tomography (PET), and functional magnetic resonance imaging (fMRI), suggest that patients with AN exhibit functional abnormalities in the frontal, parietal, and cingulate cortices [3-5]. In addition, a recent fMRI study in an adolescent population reported that activation of the medial prefrontal cortex during performance of a theory of mind task was lower in patients with AN than in controls (CTLs) [6]. These results may indicate that decreased activation of the prefrontal cortex underlies neural malfunction related to social cognition and behavior.

Although patients with ED with extremely low body weight often pose a serious clinical problem, there are few studies of this population. In addition, patients with ED require careful physical management and behavioral suppression in the acute phase of therapy. The portability, compactness, and non-invasive features of near-infrared spectroscopy (NIRS) make it an ideal tool with which to study functional brain activity in patients with ED. NIRS allows the measurement of functional brain activity under near-natural conditions [7]. It is based on the principle that near-infrared light is preferentially absorbed by oxygenated hemoglobin (oxy-Hb) and de-oxygenated hemoglobin (deoxy-Hb) compared with other body tissues [8]; furthermore, it quantifies regional oxy- and deoxy-Hb concentrations with a high time resolution. Patients with depression, schizophrenia, and bipolar disorder have been studied using NIRS, and their characteristic time courses of oxy-Hb changes in the frontal lobe have been investigated [8-10]. Four NIRS studies in patients with ED have been conducted [11-14], three of which reported lower oxy-Hb concentrations in the frontal cortex during the letter fluency task (LFT) in patients with ED than in healthy CTLs [11-13]. However, the patient population and methodology varied across these studies. For example, Uehara et al. (2007) did not evaluate the relevance of clinical symptoms [11], Suda et al. (2010) excluded patients with body mass index (BMI) less than 14.5 kg/m^2^ to exclude the effect of malnutrition [12], Nagamitsu et al. (2011) studied children [13], and Sutoh et al. (2013) studied patients with AN with relatively high BMI (mean ± SD, 17.0 ± 3.1 kg/m^2^) [14].

Some patients with ED experience feelings of social self-doubt and unhappiness, which may have implications for treatment [15]. Many studies have reported that social skills and interpersonal difficulties are strongly associated with the psychopathology of patients with ED [16,17]. It is speculated that patients with ED tend to have interpersonal sensitivity, low self-esteem, social anxiety, poor emotional support, and social inhibition, all of which are associated with ED psychopathology [16]. A recent study reported that patients with AN showed impaired cognitive flexibility as well as hypo-activity in the ventrolateral prefrontal cortex [18]. Furthermore, patients with ED had alterations in the frontal cortex that contribute to reward and anxiety processing [19]. Moreover, it was shown that the frontal cortex is involved in reward-guided learning and decision-making [20], while the reward system is associated with prosocial behavior (i.e., helping, sharing, donating, cooperating, and volunteering) [21]. For these reasons, it is hypothesized that patients with ED, especially ones with severe weight loss, have a neural abnormality that influences social cognition and behavior and prevents them from adapting well to society.

In summary, there are several studies showing that patients with AN have both feelings of social insecurity (SI) and functional abnormalities in the frontal cortex that are associated with prosocial behavior. Therefore, the correlation between SI and frontal activity may be altered in patients with AN compared to CTLs. Although a previous study showed that the correlation between frontal cortex oxy-Hb concentrations during the LFT and Eating Attitudes Test scores differed between the ED and CTL groups [13], no neuroimaging studies have directly examined the relation between frontal cortex activity and SI in subjects with ED and CTLs. The aim of the present study was to investigate brain activity and its association with social relationships in patients with ED with extremely low body weight. We tested the hypothesis that frontal cortex oxy-Hb concentrations during the LFT would be lower in patients with ED than in healthy subjects, and that the correlation between frontal cortex oxy-Hb concentrations during the LFT and Eating Disorder Inventory-2 (EDI-2) score, which includes the SI subscale, would differ between groups.

## Methods

### Participants

Twenty patients with ED and 31 healthy CTLs were included in this study (Table 1). Patients with ED were primarily recruited from November 2010 to May 2012 from among inpatients at Nagoya University Hospital. They were diagnosed in accordance with the criteria of the Diagnostic and Statistical Manual of Mental Disorders, 4^th^ edition, Text Revision (DSM-IV-TR, American Psychiatric Association, 2000). The Structured Clinical Interview for DSM-IV Axis I Disorders (SCID-I), module H, was used to define subtypes of ED. All patients with ED were treated with medication, supportive psychotherapy, and behavioral therapy during their regular hospitalization. We excluded subjects who were left-handed, male, diagnosed with bulimia nervosa (BN), and younger than 17 years, as well those who did not consent to study participation or those who did not meet the diagnostic criteria for ED as shown by findings on SCID-I. All patients with ED had food intake between 400 and 800 kcal within 4 hours before NIRS measurements.

**Table 1:** Demographic characteristics of study participants

	**ED (*n*** **= 20)**	**CTL (*n*** **= 31)**	***p***	**Cohen’s** ***d***
Age (years)	28.7 ± 7.5	29.2 ± 7.8	0.824	0.06
Education (years)	14.4 ± 2.0	15.6 ± 1.8	0.025	0.66
Number of hospitalizations	3.6 ± 4.8	N/A	N/A	N/A
Number of days of hospitalization (days)	20.6 ± 17.2	N/A	N/A	N/A
Cumulative number of days of hospitalization (days)	123.3 ± 191.5	N/A	N/A	N/A
Current BMI (kg/m^2^)	14.0 ± 2.3	21.8 ± 3.7	< 0.001	2.45
BDI score	24.1 ± 10.5	4.3 ± 4.8	< 0.001	2.58
Task performance of LFT	15.5 ± 5.2	12.6 ± 3.5	0.024	0.66

Data are expressed as mean ± standard deviation.

ED, eating disorder; CTL, control; N/A, not applicable; BMI, body mass index; BDI, Beck Depression Inventory; LFT, letter fluency task.

CTL subjects were all female and were interviewed using the SCID-I to confirm the absence of psychiatric disorders. All participants were right-handed as indicated by the Edinburgh Handedness Inventory score [22].

The ethics review committees at Nagoya University Graduate School of Medicine and Nagoya University Hospital approved the study protocol, and written, informed consent was obtained from all participants prior to enrollment.

### Assessment of clinical symptoms: eating disorder inventory-2

ED symptoms were assessed in all participants using the Japanese version of the EDI-2 [23]. The EDI-2 is one of the most frequently used, self-reported assessment instruments [24]. The Japanese version of the EDI-2 is validated and reliable tool for the evaluation of the psychopathology of ED [23]. It consists of 91 items with 11 subscales and is designed to assess attitudinal and behavioral dimensions relevant to both AN and BN. Three subscales (drive for thinness, bulimia, and body dissatisfaction) relate to the diagnosis of ED, and eight subscales (ineffectiveness, perfectionism, interpersonal distrust, interceptive awareness, maturity fears, asceticism, impulse regulation, and SI) relate to the general psychopathology of ED. The SI subscale was added to the Eating Disorder Inventory (EDI-1) [25] when it was revised to the EDI-2 [26]. SI assesses the beliefs that social relationships are tense, insecure, disappointing, unrewarding, and generally of poor quality [26].

### Assessment of clinical symptoms: beck depression inventory

Depressive symptoms were also assessed using the Japanese version of the Beck Depression Inventory (BDI) [27]. BDI scores were used to control for the role that depressive symptomatology may have on the relation between NIRS measures and SI scores.

### Activation task

The LFT was performed during NIRS measurements. LFT generally activates frontal brain areas [28], and has been used in the study of many psychiatric disorders, including ED [11-14]. Participants sat opposite a video monitor in a comfortable chair with their eyes open in a moderately lit room. Cued by instructions on the monitor, they attempted to verbally generate as many words as possible with the initial Japanese syllable of either ‘a’, ‘ki’, or ‘ha’ in 20 s. Duplications and proper nouns were not permitted. The three initial syllables were used consecutively in the above-mentioned order, and the total duration of the LFT was 60 s. The number of words generated was recorded as a measure of LFT performance. The task was performed in a block design that consisted of a 30 s pre-task baseline, 60 s LFT, and 70 s post-task baseline. During the pre- and post-task baseline periods, participants were instructed to repeat the syllables ‘a’, ‘i’, ‘u’, ‘e’, and ‘o’ [12]. Participants were able to practice the task until they fully understood the instructions before NIRS measurements began.

### NIRS measurements

**Figure 1:**
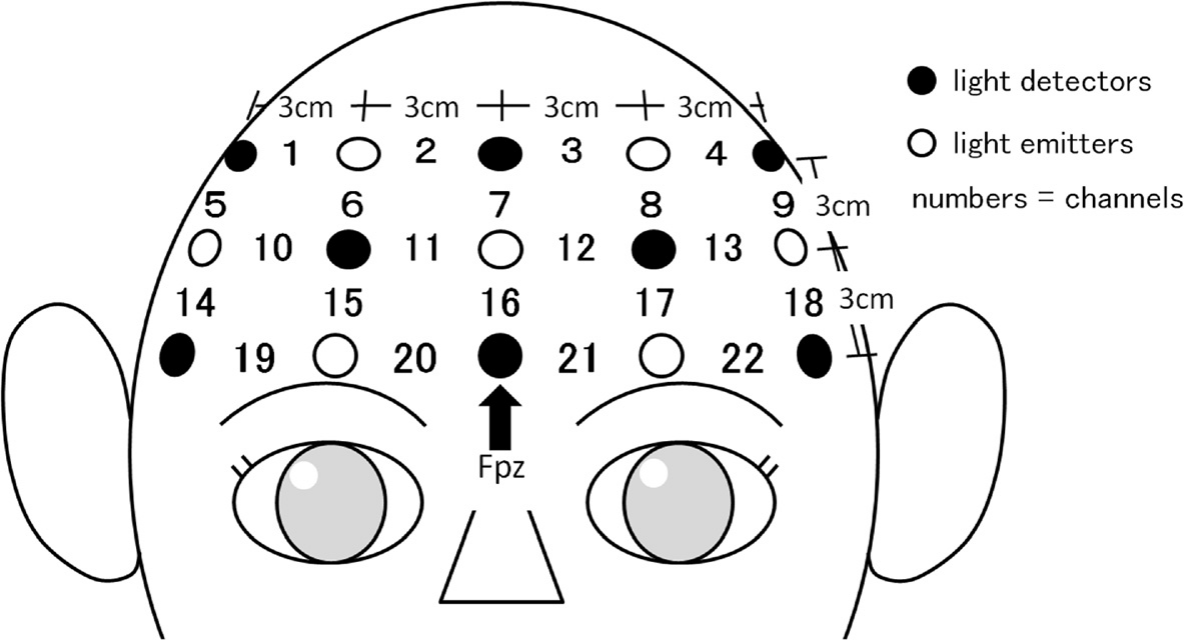
**Placement of near-infrared spectroscopy probes and channels.** The location of the light detector between channels 20 and 21 (indicated by black arrow) corresponds to FPZ of the electroencephalography 10–20 system.

Relative concentrations of oxy- and deoxy-Hb were measured at three wavelengths (780, 805, and 830 nm) by a functional NIRS system (FOIRE-3000; Shimadzu Corporation, Kyoto**,** Japan). Light emitters and detectors were arranged in an NIRS shell in a 3 × 5 array with 3-cm inter-probe distance (Figure 1). The relative concentrations of oxy- and deoxy-Hb were measured at 22 points at a depth of 2–3 cm [29] from the scalp, over a 9 × 15 cm area (Figure 1). The NIRS shell was placed over the frontal region, which was determined according to the International 10–20 system used in electroencephalography, with the lowest probes positioned along the Fp1-2 line [30]. Although the NIRS system is able to measure three kinds of hemoglobin (oxy-, deoxy-, and total-Hb), the change in oxy-Hb concentration was selected for analysis because it is the best indicator of the change in regional cerebral blood flow [31,32]. As the Hb concentration associated with changes in regional cerebral blood flow reflects changes in neuronal activity in real time [31], we used the words “high (or low) brain activity” interchangeably with “high (or low) oxy-Hb concentration”. The correspondence between the NIRS channels and the measurement points in the cerebral cortex was confirmed using virtual registration [33] based on probabilistic registration [34]. The anatomical label of the cerebral cortex was identified according to the Brodmann area [35].

### Data analysis and statistics

Oxy- and deoxy-Hb concentrations were measured in all 22 NIRS channels with a time resolution of 0.1 s. Motion artifacts were determined by close observation of the participant and the oxy-Hb waveform and were excluded from further analysis. Data were filtered with a low-pass filter at 0.1 Hz to remove short-term motion artifacts. Pre-task baseline oxy-Hb concentration was quantified as the arithmetic mean oxy-Hb concentration over the final 10 s of the 30 s pre-task baseline period. The baseline value was subtracted from the mean oxy-Hb concentration over the 60-s task period in order to determine the mean change in oxy-Hb concentration during the LFT. Also, the baseline value was subtracted from the mean oxy-Hb concentration over the 70-s post-task period to determine the mean change in oxy-Hb concentration during the post-task period.

The mean change in oxy-Hb concentration was calculated in this manner for each channel, and then compared between the ED and CTL groups using independent *t-*tests. At that time, significance levels were corrected for multiple comparisons using the false discovery rate method [36] in which the number of comparisons were 22, and effect sizes were calculated using Cohen’s *d*[37]. The mean change in oxy-Hb concentration in each channel during the LFT was correlated with the score of each subscale of the EDI-2 using Pearson product–moment correlation coefficient (r). Guilford (1956) [38] describes correlations of 0.20 > to 0.40 as “weak correlations”, correlations of 0.40 to 0.70 as “moderate correlations”, and 0.70 to 0.90 as “strong correlations, marked relationship”. Values of *p* < 0.05 were considered statistically significant for all analyses. As oxy-Hb concentrations measurements yield 22 results based on readings from 22 NIRS channels, we applied the false discovery rate method to avoid a multiple testing problem. A nominal p value was adjusted so that the adjusted p values could be compared to the same significance level of 0.05. Pearson product–moment correlation coefficient was also used to evaluate the relationship between the mean change in oxy-Hb concentration in each channel during the LFT, and age, years of education, BMI, BDI score, and LFT performance. After adjusting for variables with a significant Pearson product–moment correlation coefficient correlation, a partial correlation analysis adjusting for age, years of education, BMI, and BDI score was performed to examine the relationship between the mean change in oxy-Hb during the LFT and the score of each subscale of the EDI-2. Then, significance levels were also corrected for multiple comparisons using the false discovery rate method. All statistical analyses were performed using SPSS-20 software (SPSS Inc., Chicago, Illinois).

## Results

### Patient characteristics

All patients with ED were female and diagnosed with: anorexia nervosa binge-eating/purging type ED (13 patients), anorexia nervosa restricting type ED (4 patients), or ED not otherwise specified (EDNOS, 3 patients). All three patients with EDNOS had BMI <14.0 kg/m^2^, and did not meet the criteria of “Intense fear of gaining weight or becoming fat, even though underweight” or other criteria for BN. They corresponded to a variant termed “non-fat-phobic AN” (NFP-AN), but NFP-AN has not been recognized as a distinct diagnosis [39]. BMI at the time of NIRS measurements was significantly lower in the ED group than in the CTL group (Table 1). The number of years of education was significantly higher in the CTL group than in the ED group (Table 1). BMI at the time of NIRS measurements was significantly lower in the ED group than in the CTL group (Table 1). Information about comorbidities and medications received by patients with ED are described in an additional table (see Additional file 1).

### LFT performance, BDI, and EDI-2 score

LFT performance was significantly better in the ED group than in the CTL group (Table 1). BDI scores and the scores on all subscales of the EDI-2, including the SI subscale, were significantly higher in the ED group than in the CTL group (Table 2).

**Table 2:** Participant scores on each subscale of the eating disorder inventory-2

	**ED (*n*** **= 20)**	**CTL (*n*** **= 31)**	***p***	**Cohen’s** ***d***
Drive for thinness	9.0 ± 5.7	3.7 ± 4.0	< 0.001	1.09
Bulimia	5.7 ± 6.5	1.1 ± 1.7	0.006	1.06
Body dissatisfaction	13.8 ± 5.3	8.9 ± 6.5	0.007	0.81
Ineffectiveness	14.2 ± 6.7	4.4 ± 3.1	< 0.001	1.99
Perfectionism	5.8 ± 3.8	1.1 ± 1.7	< 0.001	1.72
Interpersonal distrust	8.3 ± 4.6	3.4 ± 2.9	< 0.001	1.31
Interoceptive awareness	11.9 ± 7.7	1.4 ± 2.3	< 0.001	2.00
Maturity fears	10.4 ± 5.0	3.5 ± 2.8	< 0.001	1.78
Asceticism	7.6 ± 5.3	3.6 ± 2.2	0.008	1.05
Impulse regulation	10.3 ± 7.5	1.3 ± 2.3	< 0.001	1.74
Social insecurity	11.3 ± 4.3	5.0 ± 3.2	< 0.001	1.69

Data are expressed as mean ± standard deviation.

ED, eating disorder; CTL, control.

### Mean change in oxy-Hb during the LFT and the post-task period

**Figure 2:**
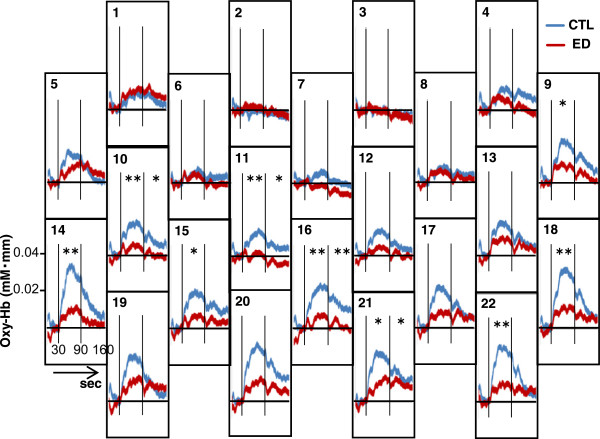
**Grand averaged waveforms of oxygenated hemoglobin concentration (Oxy-Hb) during the 60-s letter fluency task.** Blue lines represent the control group (CTL) and red lines represent patients with eating disorders (ED). Vertical lines indicate the beginning and end of the letter fluency task. *p < 0.05 and **p < 0.01 between Oxy-Hb in the ED group and Oxy-Hb in the CTL group in the task period or the post-task period, as assessed by independent *t*-test and before correction by the false discovery rate method.

Figure 2 shows the grand averaged waveforms of oxy-Hb during the LFT in each of the 22 NIRS channels. Although the oxy-Hb concentration increased as soon as the task began and decreased after the task was over in both groups, the change in oxy-Hb concentration was larger in the CTL group than in the ED group (Figure 2). The mean change in oxy-Hb concentration during the LFT was significantly greater in the CTL group than in the ED group in nine channels (Channel 9–11, 14–16, 18, 21, and 22, all *p* < 0.05, Cohen’s *d* = 0.59 to 0.96), and remained significantly greater after significance levels were corrected by the false discovery rate method in six channels (Channel 10, 11, 14, 16, 18 and 22, all *p* < 0.01, Cohen’s *d* = 0.77 to 0.96). An additional table shows the *p* values without and with correction by the false discovery rate method (see Additional file 2). According to Tsuzuki et al. (2007) [33], NIRS channels 11, 12, 15–17 and 19–22 include the bilateral orbitofrontal cortex (OFC), We found that the mean changes in oxy-Hb concentration during the LFT in NIRS channels 11, 16, 18, and 22, which include the bilateral OFC, were significantly smaller in the ED group than in the CTL group even after correction of significance levels by the false discovery rate method (all *p* < 0.05, Cohen’s *d* = 0.77 to 0.96,). Differences between the ED group and the CTL group in NIRS channels 12, 15, 17, and 19–21, which include the bilateral OFC, were not significant but did present a medium-to-large effect size (all *p* > 0.05, Cohen's *d* = 0.50 to 0.70) (see Additional file 2).

The mean change in oxy-Hb concentration during the post*-*task period was significantly greater in the CTL group than in the ED group in four channels (Channel 10, 11, 16, and 21, all *p* < 0.05, Cohen’s *d* = 0.66 to 0.78), but this difference did not remain significant after correction of significance levels by the false discovery rate method (all *p* > 0.05). Differences between the ED group and the CTL group in NIRS channels 17, 19, 20, and 22 that include the bilateral OFC did present a small effect size (Cohen’s *d* = 0.02 to 0.32) (see Additional file 3).

### Correlation between oxy-Hb concentration and demographic characteristics

In the ED group, the mean change in oxy-Hb concentration during the LFT in channels 1–3, 5, 6, 10, 13, and 17–20 significantly correlated with age (*r* = -0.46 to -0.75, all *p* < 0.05), the mean change in channel 13 significantly correlated with LFT performance (*r* = 0. 47, *p* < 0.05), and the mean change in channels 3 and 5 significantly correlated with BDI score (*r* = -0.50 and -0. 59, *p* < 0.05 and *p* <0.01, respectively). Years of education and BMI did not correlate with the mean change in oxy-Hb concentration during the LFT in any channel. Only age remained significantly correlated with the mean change in oxy-Hb concentration during the LFT in channels 1 and 5 after significance levels were corrected by the false discovery rate method (*r* = -0.64 and -0.75, *p* < 0.05 and *p* <0.01, respectively). More detailed information is shown in an additional table [see Additional file 4].

### Correlation between oxy-Hb concentration and clinical characteristics

In the CTL group, the mean change in channels 2–4 and 7 significantly correlated with BDI score (*r* = -0.39 to -0.43, *p* < 0.05). Age, years of education, BMI, and LFT performance did not correlate with the mean change in oxy-Hb concentration during the LFT in any channel. There were no significant correlations in oxy-Hb concentration during the LFT with BDI score after significance levels were corrected by the false discovery rate method (all *p* > 0.05). An additional table shows this in more detail [see Additional file 5].

**Figure 3:**
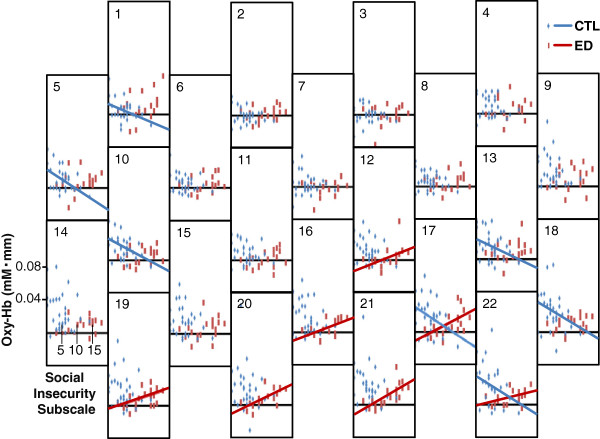
**Relation between the social insecurity subscale and the change in oxygenated hemoglobin concentration (Oxy-Hb).** Oxy-Hb during the letter fluency task was measured by each near-infrared spectroscopy channel for the control group (CTL; blue) and patients with eating disorders (ED; red). Blue and red lines represent significant correlations in the CTL and ED groups respectively.

**Figure 4:**
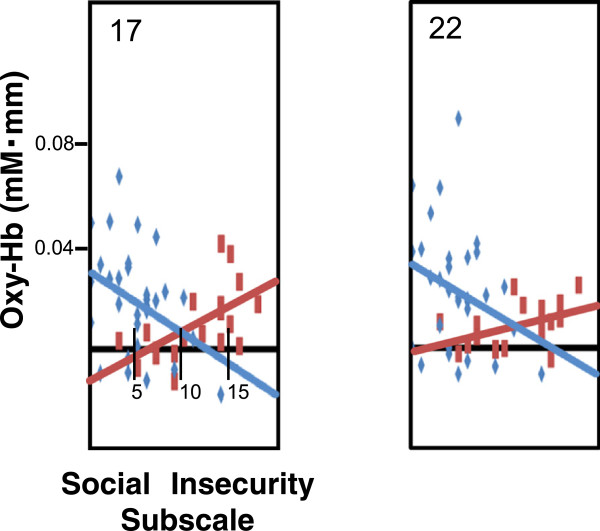
Enlarged graphs for channels 17 and 22.

In both the ED group and the CTL group, the mean change in oxy-Hb concentration during the LFT was significantly correlated with the score on the SI subscale of the EDI-2 before significance levels were corrected by the false discovery rate method (Figures 3 and 4); however, the direction of the relation was opposite in the two groups. In the ED group, the mean change in oxy-Hb concentration during the LFT in channels 12, 16, 17, and 19–22 was positively correlated with SI score (*r* = 0.47 to 0.70, all *p* < 0.05), whereas in the CTL group, the mean change in oxy-Hb concentration during the LFT in channels 1, 5, 10, 13, 17, 18, and 22 was negatively correlated with SI score (*r* = -0.38 to -0.56, all *p* < 0.05). Additional tables show these findings in more detail (see Additional files 6 and 7).

In the ED group, the mean change in oxy-Hb concentration during the LFT in channel 18 was significantly correlated with the score on the drive for thinness subscale of the EDI-2 (*r* = 0.67, *p* < 0.01), and the mean change in channel 12 was significantly correlated with the score on the bulimia subscale of the EDI-2 (*r* = 0.56, *p* < 0.05). In the CTL group, the mean change in oxy-Hb concentration during the LFT in channel 9 was significantly correlated with the score on the drive for thinness subscale of the EDI-2 (*r* = -0.39, *p* < 0.05), and the mean change in channel 4 was significantly correlated with the score on the bulimia subscale of the EDI-2 (*r* = -0.46, *p* < 0.05).

In the ED group, the correlation between the mean change in oxy-Hb concentration during the LFT and the SI score remained significant after the partial correlation analysis in channels 16, 17, and 19–22 (partial correlation coefficient = 0.62 to 0.88, *p* < 0.05), but was not significant in channel 12 (*p* > 0.05). Then significance levels were corrected by the false discovery rate method, and the correlation between the mean change in oxy-Hb concentration during the LFT and the SI score remained significant only in channel 20 and 21 (partial correlation coefficient = 0.84 and 0.88, *p* < 0.05 and *p* < 0.01, respectively). An additional table shows this in more detail (see Additional file 6). The correlation between the mean change in oxy-Hb concentration during the LFT and the drive for thinness subscale and the bulimia subscale score were not significant after the partial correlation analysis in channels 18 and 12 respectively.

In the CTL group, the correlation between the mean change in oxy-Hb concentration during the LFT and SI score remained significant after the partial correlation analysis in channels 5 and 10 (partial correlation coefficient = -0.61 and -0.44 respectively, *p* < 0.05), but was not significant in channels 18 and 20–22 (*p* > 0.05). After correction of significance levels by the false discovery rate method, the correlation between the mean change in oxy-Hb concentration during the LFT and SI score in the CTL group was not significant (all p > 0.05) (see Additional file 7).

## Discussion

### Mean change in oxy-Hb during the LFT and the post-task period

In the present study, we examined frontal cortex activity in patients with ED with extremely low body weight using hemodynamic changes measured by NIRS during an LFT. Although mean change in oxy-Hb concentration during LFT in several channels, including the bilateral OFC, did not show significant differences between the ED group and the CTL group, their effect sizes were medium-to-large. This could be due to a low statistical power to detect an effect when one is present (type II error), which is in part explained by adjustment for multiple testing and small sample size. This result suggests that the oxy-Hb concentration during LFT in the bilateral OFC tended to be lower in the ED group than in the CTL group. The result that oxy-Hb concentration during LFT in the bilateral OFC tended to be lower in the ED group than in the CTL group is consistent with our first hypothesis and previous reports [11-13].

Nagamitsu et al. [13] suggested that impairment of regional cerebrovascular reactivity might be caused by prolonged starvation or abnormal eating behavior during the illness, and that the unchanged or less fluctuating response pattern of oxy-Hb in the prefrontal area might indicate abnormal cortical processing during cognitive activation. In the following sections, we focus on the relevance of the activity of the OFC and LFT performance and EDI-2 score to discuss whether this suggestion is consistent with our results or not.

In terms of the post-task period, we cannot compare our results with a previous report directly because the previous report use different methods for data processing called linear fitting [13]. In our study, there were no significant differences in the mean change in oxy-Hb concentration during the post-task period between the ED group and the CTL group after correction of significance levels by the false discovery rate method, and their effect sizes were small to large. It is difficult to evaluate these results because of a lack of robustness.

### LFT performance, BDI, and EDI-2 score

All subscales of the EDI-2 were significantly higher in the ED group than in the CTL group, which is consistent with a previous report except for the subscale of Body Dissatisfaction [40]. Our finding that the ED group showed significantly higher scores on the BDI than the CTL group is also consistent with a previous report [41]. Several studies reported that LFT performance was significantly positively related to level of education [42-47], and that the performance of LFT fell in subjects with damage to the frontal lobe [48,49]. It is conflicting that the ED group in our study showed significantly higher LFT performance than the CTL group despite the fact that years of education were significantly lower, and oxy-Hb concentration of the bilateral OFC during the LFT tended to be smaller in the ED group than in the CTL group.

Previous NIRS reports that adopted a similar LFT protocol to our study showed that the ED group had lower frontal oxy-Hb concentration but showed almost exactly same performance on the LFT [11-13]. Nagamitsu et al. [13] mentioned that the specific patterns of oxygenation changes might indicate less supply and less demand of cerebral blood volume. A meta-analysis reported that patients with AN performed better on the LFT than CTL subjects [50]. One author suggested that this finding may be because patients with AN patients showed a higher intelligence quotient (IQ) than the CTL group [51], and LFT has been shown to have a strong relationship with IQ [52]. Even so, we cannot explain the reason that the ED group showed better performance than the CTL group on the LFT yet had a lower oxy-Hb concentration during the LFT.

Two hypotheses may help explain these findings. One hypothesis is that as a result of a malfunction in the OFC, patients with ED might have partial overactivity in other cortical brain regions such as the thalamus, parietal lobes, or temporal lobes, which are also reported to be activated during the LFT [28]. Another hypothesis is that as a result of a malfunction of the mechanisms that coordinate work and energy supply, so called “neurovascular coupling” may occur [53], and neural overactivity might occur despite low blood perfusion in the OFC in patients with ED. It was reported that in patients with ED, OFC volume was higher compared to CTL, which, in general, is supposed to reflect anxiety and high frontal activation in patients with ED [19].

Altogether, we hypothesize that overactivity in other cortical brain areas as a result of a malfunction in the OFC or neural overactivity despite low blood perfusion in the OFC might be related to high performance on the LFT and inadequate feelings of SI, for example, over-optimistic expectations. To gain evidence of the hypofrontality and better performance in patients with ED, a further study enabling measurements of the entire cortex or of neuroimaging signals of the OFC is required.

### Correlation between oxy-Hb concentration and demographic characteristics

The mean BMI of patients with ED in the present study was equivalent to the lowest BMI of patients with ED included in previous NIRS studies [11-14], and lower than the BMI of patients with ED included in many studies using fMRI [54-64] or PET [65-72]. To the best of our knowledge, this study represents the first report of both brain activity and clinical features of patients with ED with extremely low body weight. As such, the results may be influenced by malnutrition. However, the BMI of patients with ED was not significantly correlated with oxy-Hb concentration during the LFT of the bilateral dorsolateral prefrontal cortex and bilateral frontopolar areas, and the mean change in oxy-Hb concentration during the LFT was significantly correlated with SI score, even after adjusting for BMI and after correction of significance levels by the false discovery rate method. These results suggest that the BMI of patients with ED may not affect the frontal activity and SI score. Further study of recovered patients with ED is needed to examine whether the observed correlations are a trait of patients with ED that remains after recovery or are associated with the state of malnutrition.

### Correlation between oxy-Hb concentration and clinical characteristics

The mean change in oxy-Hb concentration during the LFT in channels 20 and 21, which include the bilateral OFC, had a strong, positive correlation with SI score in the ED group even after adjusting for age, years of education, BMI, and BDI score as well as correction of significance levels by the false discovery rate method. In contrast, the mean change in oxy-Hb concentration during the LFT in channels 17 and 22, which includes the left OFC, had a weak negative correlation with SI score in the CTL group before adjusting for age, years of education, BMI, and BDI score, but lacked significance after adjusting for these variables. Consistent with our hypothesis, oxy-Hb concentration during the LFT of the OFC in the ED group was lower than in the CTL group, and correlations between oxy-Hb concentration during the LFT in OFC and SI were different in the ED and CTL groups.

Recent work has emphasized the role of the OFC both in value-based decision-making [73] and in signaling outcome expectancies that are crucial for changing established behavior in the face of unexpected outcomes [74]. Signaling expected outcomes could be considered a general property of the OFC [73]. The reason that oxy-Hb concentration during the LFT in OFC correlated with only SI but not with other EDI-2 subscales may be that, in our opinion, SI is directly related to expectations and other subscales are not. The CTL group had high oxy-Hb concentration in the OFC during the LFT, and there is a tendency that the higher the oxy-Hb concentration of the OFC, the lower the SI score.

We propose both that the CTL group, which showed increased oxy-Hb concentration in the OFC during the LFT, behave adaptively and exhibit value-based decision-making in the face of unexpected outcomes in complex human relationships, and that this adaptive behavior may enable them to solve problems and form good human relationships, thus enabling them to integrate in society; as a result, their SI score becomes lower.

In contrast, the ED group had low oxy-Hb concentration in the OFC during the LFT and a high SI score, and the lower the oxy-Hb concentration of the OFC, the lower the SI score. In other words, the patients with ED who have low oxy-Hb concentration of the OFC during the LFT tend not to feel SI. We propose that low oxy-Hb concentration during the LFT of the OFC means that patients with ED neither behave adaptively nor exhibit value-based decision-making in the face of unexpected outcomes in complex human relationships, and that this maladaptive behavior may inhibit the formation of human relationships, thus isolating patients with ED from society. In support of this proposal, it has been shown that patients with ED have a non-assertive interpersonal style, greater social skill difficulties, less socially effective behavior, a smaller social support network, and more difficulties using this network than CTL subjects [16]. Furthermore, OFC malfunction may mean that patients with ED are not aware of their isolation. This is supported by a report finding that although AN patients had significantly less social support than BN patients, they were satisfied with the support they received [75]. This phenomenon may relate to denial of illness that most patients with AN have, which is also associated with resistance to treatments observed in these patients [76].

A solid therapeutic relationship is recommended to overcome treatment resistance [76,77], and it might be also recommended for patients with ED who have low oxy-Hb concentration of the OFC during the LFT. Interpersonal psychotherapy that improves interpersonal functioning by enhancing communication skills in significant relationships has been reported to be an effective therapy for AN [78].

Therefore, malfunction of the OFC may underlie the maladaptive behavior of patients with ED and may represent a biological cause of the psychopathological factors of ED. To support this hypothesis, methodological improvements that can investigate relations between the function of the OFC and performance of tasks that directly induce SI are needed in future studies. In addition, a comparison of AN and BN using the same method would be of interest because there is evidence that there are functional and structural cerebral differences between BN and AN [79].

Regional hemodynamic changes in the left dorsolateral prefrontal cortex (Channel 18) were positively correlated with the drive for thinness score, and regional hemodynamic changes in the left frontopolar area (Channel 12) were positively correlated with the bulimia score. This is inconsistent with a previous study using the Japanese version of the Eating Attitude Test (EAT-26) [80,81], which reported that regional hemodynamic changes in the right frontotemporal regions negatively correlated with dieting tendency scores on the EAT-26, and regional hemodynamic changes in the left OFC negatively correlated with binge eating scores in patients with ED [12]. These discrepancies may be due to differences in the methods used to evaluate ED symptoms and/or differences in ED patient characteristics such as BMI. Further study is needed to test these possibilities. In the present study, the higher the drive for thinness score, the larger the increase in oxy-Hb concentration in the left prefrontal cortex during LFT in the ED group. However, a previous PET study reported that [18 F]-altanserin binding potential in several cortical regions, including the prefrontal cortex, was negatively related to the drive for thinness in patients with AN [82]. It is interesting that oxy-Hb concentration increased in the left prefrontal cortex of patients with ED during LFT, whereas metabolism in the same region was decreased.

### Study limitations

This study has several limitations. First, the number of participants was small, and further study with more participants is required to increase the statistical power. There was heterogeneity of ED subtypes, but each subtype had too few participants to analyze intra-group differences. Additional subjects are needed for future studies. Second, the ED group was not homogeneous in terms of comorbidity, psychotherapy, and medications, and this may have influenced the results. Third, there may be a selection bias in the CTL group such as years of education, because they were recruited from the hospital staff. Fourth, NIRS has several methodological limitations. The exact measurement point over the cortex differs across subjects according to the size of the skull and brain; therefore, the point of measurement can only be determined in a probabilistic manner. In addition, determining the exact distance of the near-infrared light emitters from light detectors that is required to calculate the change in oxy-Hb remains difficult. As brain atrophy has been seen in patients with AN [2,83-85], data from the NIRS was possibly affected due to path length factors of near-infrared light. Moreover, evaluating deep structures of the brain is not possible. The validity of NIRS measured on the forehead as a measure of functional brain activity is unknown; however, according to Takahashi et al. (2011), NIRS signals measured on the forehead during the LFT would reflect task-related changes in subcutaneous blood flow [86].

## Conclusions

In conclusion, the present NIRS study is, to the best of our knowledge, the first report of the relationship between SI and brain activity in patients with ED with extremely low body weight. More frontal reactivity was associated with lower SI scores in the CTL group and less frontal reactivity was associated with lower SI scores in patients with ED with extremely low body weight. These results indicate that patients with ED with extremely low body weight had OFC malfunction and higher SI scores, which may underlie their lack of insight and social isolation. Further studies targeting larger samples of patients with ED, including those who have recovered, are necessary.

## Competing interests

The authors declare that they have no conflicts of interest.

## Authors’ contributions

HK, ST, and NO conceived and designed the experiments. HK, KK, ST, MI, NK, and KN performed the experiments. HK, KK, NK, MA, TI, and NO analyzed the data. YN contributed reagents/materials/analysis tools. HK, KK, NK, BA, TI, and NO wrote the paper. All authors read and approved the final manuscript.

## Pre-publication history

The pre-publication history for this paper can be accessed here:

http://www.biomedcentral.com/1471-244X/14/173/prepub

## Supplementary Material

Additional file 1 Information about comorbidities and medications received by patients with eating disorders. Click here for file

Additional file 2 Mean change in oxygenated hemoglobin concentration during the letter fluency task. Click here for file

Additional file 3 Mean change in oxygenated hemoglobin concentration during the post-task period. Click here for file

Additional file 4 Correlation between mean change in oxygenated hemoglobin concentration during the letter fluency task and letter fluency task performance, age, Beck Depression Inventory score, years of education, and body mass index in the eating disorders group. Click here for file

Additional file 5 Correlation between mean change in oxygenated hemoglobin concentration during the letter fluency task an letter fluency task performance, age, Beck Depression Inventory score, years of education, and body mass index in the control group. Click here for file

Additional file 6 Correlation between the mean change in oxygenated hemoglobin concentration during the letter fluency task and the social insecurity score in the eating disorder group. Click here for file

Additional file 7 Correlation between the mean change in oxygenated hemoglobin concentration during the letter fluency task and the social insecurity score in the control group. Click here for file
